# Harnessing the Power of Optical Microscopic and Macroscopic Imaging for Natural Products as Cancer Therapeutics

**DOI:** 10.3389/fphar.2019.01438

**Published:** 2019-11-28

**Authors:** Tianyu Yan, Qi Zeng, Lin Wang, Nan Wang, Honghao Cao, Xinyi Xu, Xueli Chen

**Affiliations:** ^1^Engineering Research Center of Molecular and Neuro Imaging of Ministry of Education and School of Life Science and Technology, Xidian University, Xi’an, China; ^2^School of Information Sciences and Technology, Northwest University, Xi’an, China

**Keywords:** natural products, optical imaging, microscopic imaging, macroscopic imaging, cancer

## Abstract

Natural products (NPs) are an important source for new drug discovery over the past decades, which have been demonstrated to be effectively used in cancer prevention, treatment, and adjuvant therapy. Many methods, such as the genomic and metabolomic approaches, immunochemistry, mass spectrometry, and chromatography, have been used to study the effects of NPs on cancer as well as themselves. Because of the advantages in specificity, sensitivity, high throughput, and cost-effectiveness, optical imaging (OI) approaches, including optical microscopic imaging and macroscopic imaging techniques have also been applied in the studies of NPs. Optical microscopic imaging can observe NPs as cancer therapeutics at the cellular level and analyze its cytotoxicity and mechanism of action. Optical macroscopic imaging observes the distribution, metabolic pathway, and target lesions of NPs *in vivo*, and evaluates NPs as cancer therapeutics at the whole-body level in small living animals. This review focuses on the recent advances in NPs as cancer therapeutics, with particular emphasis on the powerful use of optical microscopic and macroscopic imaging techniques, including the studies of observation of ingestion by cells, anticancer mechanism, and *in vivo* delivery. Finally, we prospect the wider application and future potential of OI approaches in NPs as cancer therapeutics.

## Introduction

As an important tool to break through the bottleneck of drug development, natural products (NPs) and their derivatives have contributed about 50% of new drugs over the past 30 years (Camp et al., 2012; Newman and Cragg 2016), including anti-cancer, anti-inflammatory, and antibiotics (Shukla and Singh 2011). NPs extracts and some herbal formulas have proven to be useful in the prevention and treatment of cancer (Zhang et al., 2018). In China, the addition of traditional Chinese medicines or Chinese herbal medicines derived from NPs to cancer treatment is being accepted by more and more people because of its ability to improve the quality of life and low toxicity (Kasymjanova et al., 2018). NPs have the following effects in anti-cancer. Firstly, certain NPs can be used to prevent cancer, such as *Urtica dioica*, an edible plant with anticancer ability (Esposito et al., 2019). Secondly, NPs that have been confirmed to be toxic to cancer cells may be directly used in the treatment of cancer in the future. For example, isoflavones have effective growth inhibition and apoptosis induction effects on human and animal cancer cells (Sarkar and Li, 2009), and ursolic acid has anti-cancer properties for breast cancer and colorectal cancer (Chan et al., 2019). Thirdly, NPs can be used in combination with radiotherapy and chemotherapy. It has been reported that resveratrol can be used as an effective chemical protection and synergistic agent in cancer chemotherapy (Zhang et al., 2018; Xiao et al., 2019). Some cancers may not be sensitive to radiotherapy and chemotherapy, such as pancreatic cancer. In this case, NPs of various components can improve their therapeutic effect (Yue et al., 2017). In addition, multidrug resistance of tumors limits the therapeutic effects of existing antitumor drugs (Guo et al., 2017). Therefore, obtaining new anticancer drugs from NPs is an important method for humans to resist drug toxicity, drug resistance, and improve targeting (Saha and Khuda-Bukhsh, 2013).

At present, there are a variety of techniques available as a research method for obtaining anticancer drugs from NPs. First of all, functional genomics can study natural drugs from the perspective of mechanism of action (Harvey et al., 2015). Lukasz Huminiecki et al. have reviewed published functional genome studies on the relationship between curcumin and cancer, revealing the anticancer process of curcumin (Huminiecki et al., 2017). Zhenyu Yue et al. have developed a machine learning method that can comprehensively predict the response of NPs to a group of cancer cell lines based on the gene expression and chemical properties of NPs (Yue et al., 2015). Secondly, metabolomics methods can be used for quantitative and qualitative metabolite assessments in biological systems for environmental toxicology analysis and disease diagnosis (Cox et al., 2014). Vittoria Graziani et al. analyzed the cytotoxic effects of 31 metabolites from 14 legumes on colon cancer cell lines by metabolomics (Graziani et al., 2018). Tawfike et al. used metabolomics tools to analyze the effect of bioactive metabolites of *Curvularia* extract against leukemia cell line of K562 (Tawfike et al., 2018). Thirdly, chemical proteomics methods can provide important clues in the study of molecular targets of NPs (Yue et al., 2012). Yiqing Zhou et al. used a NP of pseudolaric acid B derived photoaffinity probe to directly target CD147, a glycosylated transmembrane protein on the surface of tumor cells, by chemical proteomics method (Zhou et al., 2017). Haibin Shi et al. developed a cell-permeable kinase probe derived from staurosporine for proteomic analysis of potential cellular targets in HepG2 cells (Shi et al., 2011). These methods mentioned above require some special tools such as chromatography, mass spectrometry, and Western blotting (Wu and Liang, 2010; Yang et al., 2015; da Silva et al., 2018; Wu et al., 2018).

However, methods such as genomics and metabolomics do not provide an intuitive morphological or functional image, while optical imaging (OI) techniques can compensate for this deficiency, providing two-dimensional or three-dimensional spatial distribution and functional information of drugs and lesions at the microscopic and macroscopic scales (Krucker and Sandanaraj, 2011; Walsh et al., 2017; Song et al., 2019). OI technology is a high-throughput detection technology. Due to its advantages in time and spatial resolution, imaging sensitivity, tissue specificity (Ntziachristos et al., 2005), OI technology has been widely used in gene expression, substance metabolism, cancer detection, drug development, and other fields (Weissleder et al., 1999; Sharpe et al., 2002; Gao et al., 2005; Sega and Low, 2008). OI covering microscopic and macroscopic imaging scales, can be used for imaging or analyzing living system at different levels, including molecular, cellular, tissue, and organ levels (Moriyama et al., 2008). Therefore, in the development of NPs based anticancer drugs, OI technology can be of great applicability in studying composition and action mechanism of drugs as well as evaluating their therapeutic effects. Optical microscopic imaging having a spatial resolution at micron or sub-micron level is suitable for observation of morphological and subcellular structures of cells, as well as quantitative analysis of biochemical components inside the cells (Gordon et al., 2007; Cui et al., 2008). With the help of super resolution techniques, molecular structures with resolutions below the diffraction limit (i.e., < 200 nm) can be achieved (Bullen, 2008). Optical macroscopic imaging technology can achieve large-scale imaging with resolution of sub-millimeter scale at tissue or organ level (Walsh et al., 2017). Importantly, it can provide *in vivo* whole-body imaging of living animals (Yang et al., 2000). With the help of labeling technique, optical macroscopic imaging can be used for tracking the delivery of drugs *in vivo*, detecting the enrichment state of drugs, as well as performing specific imaging of tumors to analyze the development of diseases and evaluate the therapeutic effect of drugs (Ntziachristos et al., 2004; Walsh et al., 2017).

In this review, we focus on the contribution of OI technology as a research tool to study NPs based anticancer drugs, including the studies of structure, composition, action mode, and therapeutic effects, and so on. The OI technologies include optical microscopic imaging technology represented by fluorescence microscopy and super-resolution microscopy, and optical macroscopic imaging technology with an example of near infrared (NIR) fluorescence imaging technique having a good targeting ability and detecting depth in the *in vivo* imaging of NPs. Finally, we prospect the wider application and future potential of OI approaches in NPs as cancer therapeutics.

## Application of Optical Microscopic Imaging for Natural Products

Cell experiments of NPs by using a variety of cancer cell lines is an indispensable step in the screening of a NP with anticancer potential (Krutzik et al., 2008; Ashidi et al., 2010; Kell, 2013). Three aspects should be concerned in this process. First, the uptake of a compound derived from NPs in one cancer cell needs to be verified (Xing et al., 2012). Second, it is necessary to confirm action mechanism between NPs and cancer cells as well as the cell toxicity, which includes binding to specific organelles or cell structures, inhibiting the expression of important proteins in cells, and other factors that can cause changes in cell status (Lin et al., 2014; Cavalieri et al., 2015; Xie and Peng, 2017). Third, the effect of inducing apoptosis or inhibiting the increment of cancer cells by NPs should be investigated (Earley et al., 2012). Having high spatial resolution, optical microscopic imaging technology enables precise imaging of cell morphology and structures. Further combining with fluorescent labeling technique, it can realize the tracking and specific imaging of specific substance or structure labeled by fluorescent probes (Xie and Peng, 2018). With targeted labeling, fluorescence microscopy can monitor the NPs uptake in cells, track the intracellular targets of NPs, observe the NPs-induced destruction of cells, and count the cells having morphological changes to quantitatively analyze the anticancer effects of NPs. This high-resolution visualization provides the most direct evidence for anticancer studies of NPs.

### Observation of Natural Products Ingested by Cancer Cells

**Figure 1:**
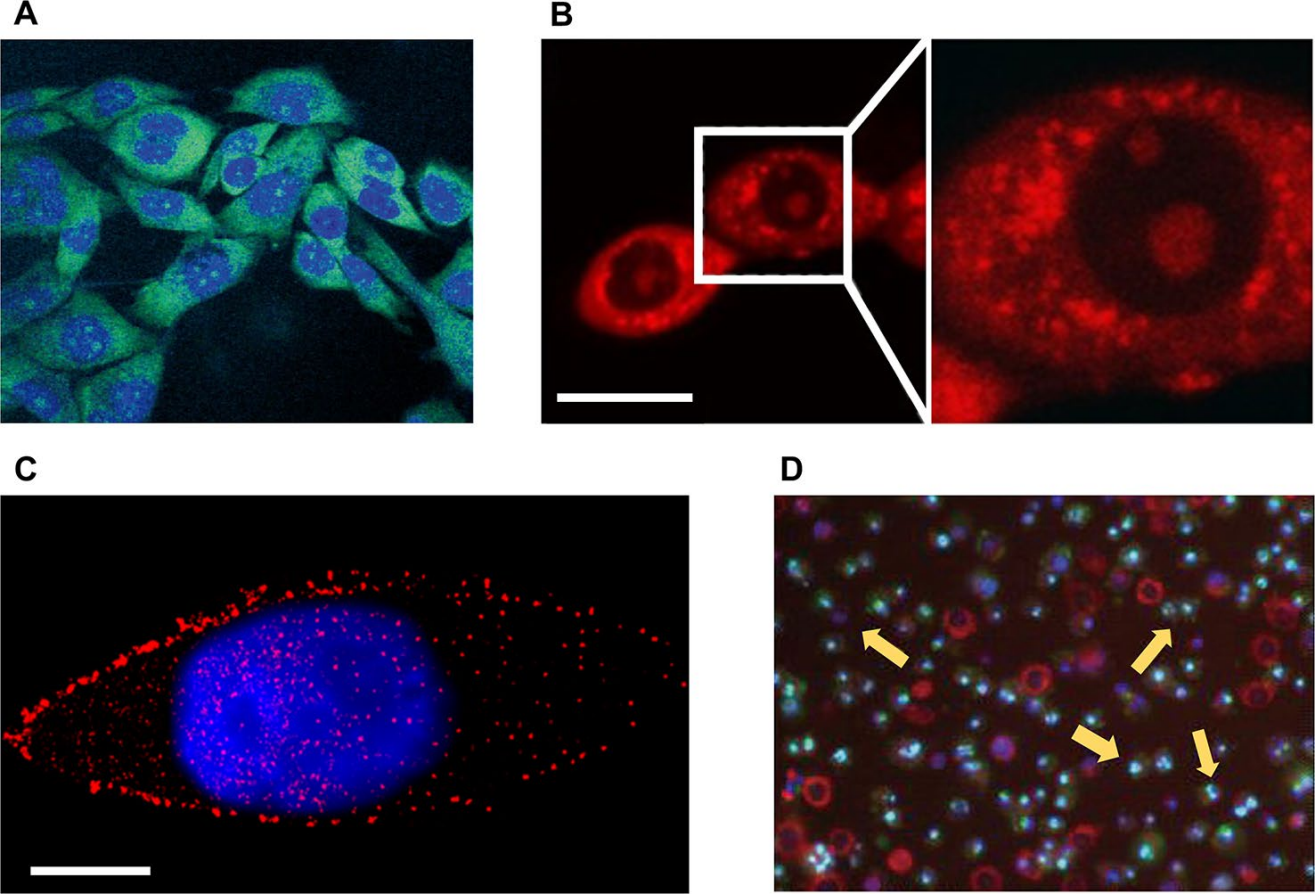
Microscopic imaging of natural products in cell experiments. **(A)** The green fluorescent 12-aza-epothilone (azathilone) derivative localization in the cytoplasm, the blue area indicates the fluorescence imaging result of nucleus. Adapted with permission from Ref. Gertsch et al., 2009. **(B)** Confocal laser scanning microscopy detects mitochondria fission and swelling. Scale bar: 20 μm. Adapted with permission from Ref. Li et al., 2015. **(C)** Direct stochastic optical reconstruction microscopy imaging mTOR (red) and cell nucleus (blue). Scale bar: 10 μm. Adapted with permission from Ref. Teng et al., 2017. **(D)** Apoptosis assay of cells treated with apigenin by flow cytometry, yellow arrows indicate apoptotic cells. Adapted with permission from Ref. Cao et al., 2013.

Fluorescence based microscopic imaging has been an important research tool in the biomedical field over the past few decades. Fluorescence microscope and super-resolution microscope which provide high-resolution images of living systems, can observe the state of NPs ingested by cancer cells (Fumagalli et al., 2015), which is helpful for the preliminary screening of anticancer activity of NPs. Furthermore, with the fluorescence based microscopic imaging techniques, the distribution of NPs components in the cells can be precisely tracked to provide evidence for the targeting of the NP. Jürg Gertsch et al. prepared a green fluorescence labeled 12-aza-epothilone (azathilone) derivative, and used confocal laser scanning microscopy (CLSM) to directly observe that it entered into cancer cells and was distributed only in the cytoplasm (**Figure 1A**). Further results demonstrated that it bound to cell microtubules and inhibited the proliferation of cancer cells by preventing cell cycle from entering G2/M conversion (Gertsch et al., 2009). Maria V. Chatziathanasiadou et al. synthesized quercetin-alanine bioconjugation based on quercetin which is cytotoxic to cancer cells, and detected its cellular internalization to observe the cytotoxicity using confocal microscopy. The results demonstrated that quercetin-alanine bioconjugation had stronger cytotoxicity, which indicated that the bioconjugates of NPs could enhance the therapeutic effects of NPs (Chatziathanasiadou et al., 2018). Fidelia I. Uche et al. used CLSM to detect intracellular uptake of cycleanine that was labeled with Alexa 488 azide *via* specific click chemistry reaction and demonstrated the resistance of cycleanine to ovarian cancer (Uche et al., 2018). For tracking the drug entering the cell, Liwei Huang et al. used liposomes modified with folic acid to deliver artemisinin into lysosomes of cancer cells, resulting in lysosomal membrane permeabilization and inducing cell death. In their study, CLSM was used to detect the distribution of artemisinin in lysosomes (Huang et al., 2017). Paolo Beuzer et al. developed a STORM (stochastic optical reconstruction microscopy, a super-resolution imaging strategy) based experimental scheme to verify the anticancer potential of ophiobolin A, a phytotoxin produced by plant pathogen *Drechslera gigantea* and labeled with small molecule based fluorescent probes. The STORM technique provided high resolution image (12 to 30 nm) of the fluorescent probes, revealing that the fluorescence signals in the high concentration region are uniformly distributed in the nucleus and cytoplasm, while most of the signals in the lower concentration region are located outside the nuclear membrane (Beuzer et al., 2016).

### Investigation of Anticancer Mechanism of Natural Products

Different NPs prevent the proliferation and induce the apoptosis of cancer cells in various ways, including disrupting the cytoskeleton, deactivating the function of a certain organelle, reducing the expression level of essential proteins, and interfering with the proliferation and division of cells. These changes often lead to significantly morphological feedback, which can be easily captured by optical microscopic imaging devices. For example, it has been reported that the optical microscopic imaging can be used to detect the cytoskeleton of cancer cells destroyed by NPs. Marisa Rangel et al. extracted geodiamolides A, B, H, and I from the marine sponge *Geodia corticostylifera* on the Brazilian coast and found that they have anti-proliferative effects on human breast cancer cell lines of T47D and MCF7. With the help of CLSM, they found that the anticancer mechanism of geodiamolides is to destroy the actin filaments in cancer cells, demonstrating the potential of these compounds to fight cancer (Rangel et al., 2006). Radim Havelek et al. used structured illumination microscopy, a super-resolution imaging strategy, to examine the effects of homochelidonine and chelidonine on blood cancer cells. Relevant results showed that homochelidonine and chelidonine caused microtubule disruption around the nucleus (Havelek et al., 2016). Optical microscopic imaging can also be used to explore effective dose of NPs. Wen-Jing Guo et al. employed CLSM to investigate the effects of different concentrations paclitaxel on the death form of human lung cancer cells. Corresponding results showed that paclitaxel of low concentration (35 nM) can lead to changes in the mode of apoptosis, including nuclear fragmentation, phosphatidylserine externalization, and G2/M cell cycle arrest. Although the mechanism of cell death induced by high concentration paclitaxel (70 µM) was not clear, imaging results showed that high concentration paclitaxel (70 µM) can lead to obvious cytoplasmic vacuolation (Guo et al., 2010). Xin Li et al. studied the mechanism of Guttiferone F on the growth inhibition of prostate cancer cells. They used red fluorescent dye to stain cells for observation of mitochondria *via* CLSM (**Figure 1B**). Results of LNCaP cells in serum depleted medium showed that the action of 10 µM Guttiferone F will cause the swelling of mitochondria, ultimately leading to cell apoptosis (Li et al., 2015). About the detection of life activities of cancer cells, Osayemwenre Erharuyi et al. reported their detection of anticancer NPs of African folklore. The CLSM results showed that one of the NPs, *Jatropha multifida* affected the cellular respiration of MCF-7 and BT-20 cells (Erharuyi et al., 2014). With the help of specially designed probes, super-resolution microscopy can be used to directly detect the concentration and distribution of specific proteins in cancer cells. Bo Teng et al. studied the molecular mechanism of anticancer effect of 20(S)-protopanaxadiol (PPD), a natural product of *ginseng*, on Hep-2 cells. With the help of direct STORM (STORM) technique, the authors demonstrated that PPD could reduce the number of mTOR as well as its downstream proteins of 4EBP1 and eIF4E (**Figure 1C**), and the degree of decrease was proportional to the concentration of PPD. Thus, PPD can inhibit mTOR pathway by reducing the expression level of related signal proteins (Teng et al., 2017).

### Verification of Apoptosis

The decrease in proliferation rate of cancer cells and the apoptosis of cancer cells can provide the most direct evidence for cancer therapeutics. Optical microscopic imaging techniques have been already employed to observe the cell apoptosis and quantitative analysis of drug-treated cancer cells, including the traditional chemotherapeutic drugs and NPs (Sharma et al., 2012). Agata Antosiak et al. studied the toxicity of genistein on human ovarian cancer cells and monitored apoptosis of cancer cells using CLSM, whose images provided morphological information of cells, including membrane blebs and condensed or pyknotic nuclei. The experimental results confirmed anticancer activity of genistein-genistein-8-C-glucoside against ovarian cancer (Antosiak et al., 2017). Xucen Cao et al. analyzed the toxicity of apigenin (4’,5,7-trihydroxyflavone, a member of the flavone subclass of flavonoids present in fruits and vegetables) on the human breast cancer cells of T47D and MDA-MB-231, and the apoptosis of cells was observed using fluorescence microscopy and flow cytometry (**Figure 1D**) (Cao et al., 2013).

## Application of Optical Macroscopic Imaging for Natural Products

*In vitro* cell experiments can observe the toxicity of NPs to different cancer cell lines, but when applied to living organisms, the complexity increases dramatically compared to the physiological environment in petri dishes (Walsh et al., 2016). Fortunately, *in vivo* experiments of small animals can establish a pharmacokinetics model closer to clinical application environment for NPs with anticancer potential (Dufort et al., 2010). Such experiments are usually performed by injecting the tested drugs into small animals implanted with tumors, which facilitates *in vivo* detection of tumors, and *in vivo* tracking the delivery as well as enrichment of drugs (Tsai et al., 2014). In this case, optical microscopic imaging technology is difficult to play an important role due to the low imaging depth and small field of view. With the help of fluorescent labeling technology, optical macroscopic imaging technology, such as fluorescence imaging technique, can be well adapted to whole body small animal imaging, providing with a large field of view and an acceptable resolution (Chen et al., 2014). By labeling the NPs with fluorescence probes, optical macroscopic imaging technology can monitor the transportation, enrichment and metabolism of drugs in the whole body of small animals *in vivo* (Bednar et al., 2007).

### Monitoring the Delivery of Natural Products *In Vivo*

**Figure 2:**
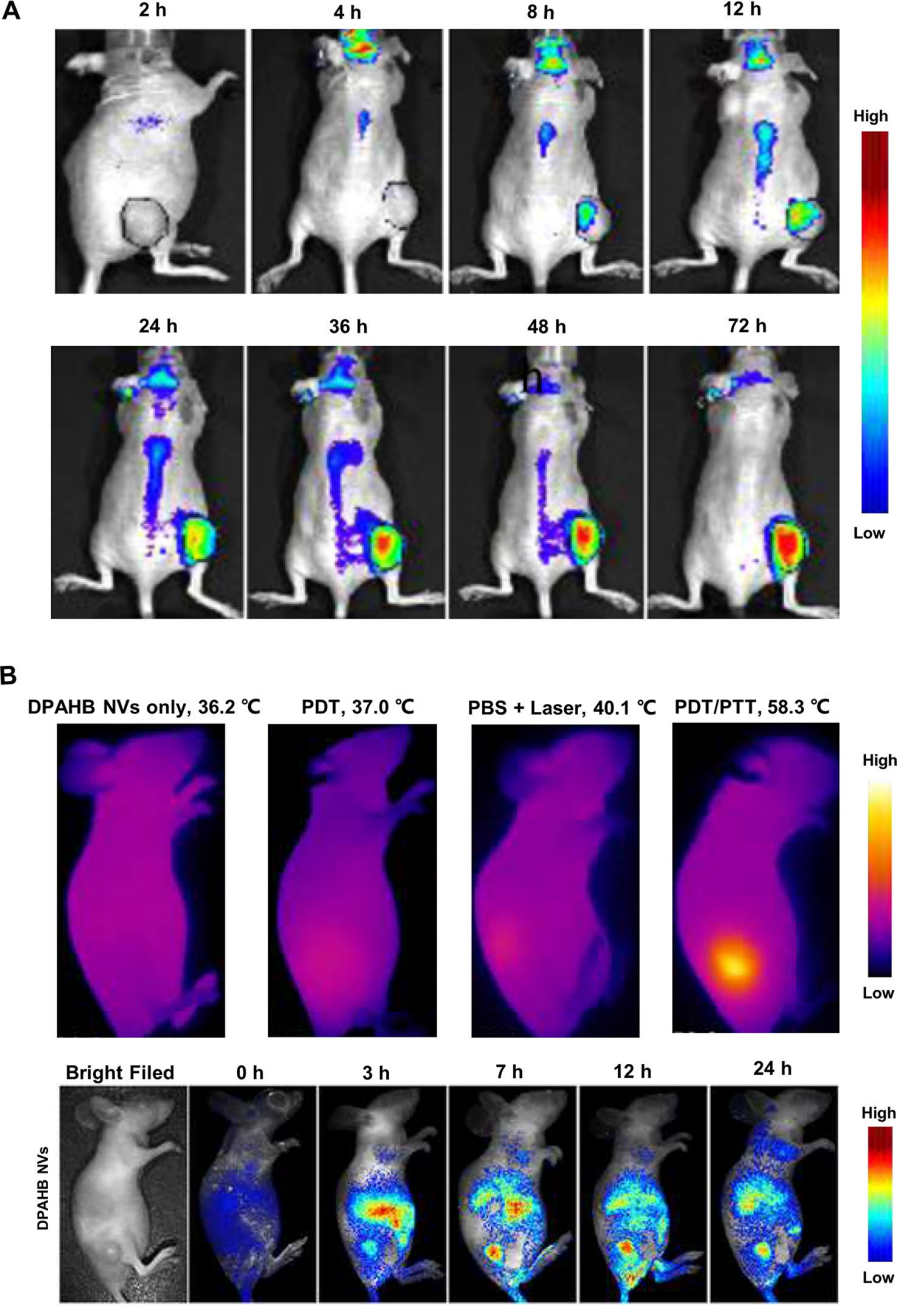
Macroscopic imaging of natural products in living animal experiments. **(A)** Delivery process of gambogic acid in nude mice implanted with HT-29 cells. Adapted with permission from Ref. Huang et al., 2015. **(B)** Fluorescence images and infrared camera imaging results show that DPAHB nanovesicles can be used for both NIR fluorescence imaging and photothermal. Adapted with permission from Ref. Zheng et al., 2018.

By conjugating the drug and fluorescent probe to a specific nano-carrier, optical macroscopic imaging technology enables real-time tracking of drug transport and enrichment within the organism with the help of fluorescent probe. Jianqin Lu et al. developed PEG_5K_-EB_2_ micelles in which the polyethylene glycol 5000 (PEG_5K_) and embelin (EB, a NP with anticancer activity) were conjugated to encapsulate hydrophobic drugs such as paclitaxel (PTX, a broad-spectrum cancer therapeutic). With the help of NIR fluorescence imaging, the enrichment of PEG_5K_-EB_2_ micelles within living animal can be observed *in vivo*, whose results showed that PEG_5K_-EB_2_ micelles mainly concentrated in the tumor site of mice except that a small amount existed in the liver and spleen (Lu et al., 2013). In order to improve the delivery capacity of gambogic acid (GA), a NP having anticancer ability, Wenzhe Huang et al. prepared a nano-formulation of GA using a series of telodendrimers and then applied it to living nude mice implanted with HT-29 cells (a human colon cancer cell line). Results of *in vivo* NIR fluorescence imaging showed that GA accumulated in tumor area (**Figure 2A**), which proved that the nano-formulation of GA had good targeting ability and a great potential of replacing traditional chemotherapy in colon cancer treatment (Huang et al., 2015).

### Combining With Natural Products to Assist Cancer Treatment

It has been reported that certain compounds of NP can be used as a heat-generating agent of photothermal therapy (PTT) for adjuvant treatment of tumors, while NIR fluorescence imaging technology has good synergy with PTT. Xiuli Zheng et al. modified the hypocrellin B isolated from traditional Chinese medicine of *Hypocrella bambusae* with 1,2-diamino-2-methyl propane to form amino-substituted hypocrellin (DPAHB), and they combined DPAHB with poly(ethylene glycol)-b-poly(lactic-co-glycolic acid) to prepared biodegradable water-dispersible nanovesicles(DPAHB NVs). Under laser irradiation, the cytoplasm of 4T1 cells incubated with DPAHB NVs produced strong NIR emission, proving that the DPAHB NVs can be successfully absorbed by cancer cells and used as a contrast agent for fluorescence imaging. NIR fluorescence imaging of 4T1 tumor-bearing nude mice intravenously injected with DPAHB NVs, showed that DPAHB NVs accumulated in tumor, liver, and kidney. Under 721 nm excitation (0.8 W cm^−2^) to perform IR thermography, results showed that the temperature of tumor site where DPAHB NVs accumulated rapidly increased to 58.3°C (**Figure 2B**), which proved that DPAHB NVs can effectively convert NIR light into heat and be used for PPT treatment of tumors (Zheng et al., 2018). In the follow-up study, the same research group constructed hypocrellin-derivative nanoparticles (APHB NPs) and applied it to the *in vivo* deep tumor imaging, in which they successfully realized the synchronization of NIR fluorescence imaging and sonodynamic therapy of cancer treatment (Zheng et al., 2019).

## Conclusion and Perspective

NPs are an important source for new drug discovery, and have been demonstrated to be effectively used in cancer prevention, treatment, and adjuvant therapy. Although many methods, such as the genomic approach, metabolomics approach, immunochemistry, mass spectrometry, and chromatography, have been used to study the effects of NPs on cancer as well as themselves, such tools do not provide an intuitive morphological or functional image. OI techniques, providing two-dimensional or three-dimensional spatial distribution and functional information of drugs and lesions at the microscopic and macroscopic scales, can compensate for this deficiency. This work reviewed the recent advances in NPs as cancer therapeutics, with particular emphasis on the powerful use of optical microscopic and macroscopic imaging techniques, including the studies of observation of ingestion by cells, anticancer mechanism, *in vivo* delivery, and therapeutic effects. With high time and spatial resolution, high sensitivity, and high specificity, OI technology has been widely used in drug development and therapy effect evaluation. In particular, some techniques have been used in clinical studies (Fung et al., 2007; Schaafsma et al., 2011). However, the potential applications of OI technology in NPs has not been fully tapped.

Screening of anticancer NPs requires accessing a variety of indicators, including the molecular structure, composition distribution, metabolic pathways, as well as the targeting, dosage, anticancer effects, and toxic side effects on cells, tissues, and living organisms. Raman spectroscopic imaging (RSI), providing the unique fingerprint information related to vibration bands of molecules, has the advantages of high chemical specificity, non-invasive detection capability, low sensitivity to water, and no special sample pretreatment, so that it has become an invaluable tool in the field of medicinal chemistry (Zhao et al., 2007; Evans and Xie, 2008; Freudiger et al., 2008; Kneipp et al., 2010). RSI has been widely used in all aspects of pharmaceuticals, including crystal form study, composition distribution and identification, pharmacological screening, and monitoring anticancer effect on cells and tissues (Popp and Windbergs, 2015; Seidel et al., 2019). Genetically modified probe based bioluminescence imaging can be used to study the metabolic pathways, anticancer mechanisms as well as toxic side effects on living small animals *in vivo* (Tung et al., 2014; Liu et al., 2015; Horibe et al., 2018). Dynamic OI techniques can *in vivo* monitor the delivery process and whole-body distribution of drugs inside the body of living animals, which is great helpful for the study of drug metabolism and mechanism of action (Moriyama et al., 2008; Ma et al., 2015; Zhang et al., 2017). The application examples of the above OI technology in medicinal chemistry can be used in the study of NPs in the same way.

During screening of anticancer NPs, a single research tool cannot achieve such comprehensive information at the same time, whether it is genomics, metabolomics, or OI technology. In addition, OI technology also has some shortcomings that cannot be ignored. For example, there is a contradictory balance among spatial resolution, field of view, and imaging speed. Penetration depth is another huge defect of OI technology, because light is easily scattered and absorbed by tissues. Fortunately, OI technology has good compatibility to integrate with other techniques, which can help it overcome the defects and expand the applications. For example, fusing with X-ray or CT imaging, OI technique can be powerfully used for imaging pharmacokinetic rates (Zhang et al., 2013), visualization of antitumor treatment (Ntziachristos et al., 2004), monitoring anticancer drug delivery (Tian et al., 2015), discriminating the efficacy of disease-modifying anti-rheumatic drug (Peterson et al., 2010), and so on. Similarly, OI techniques are also integrated with magnetic resonance imaging, nuclear imaging, or optoacoustic imaging (Ma et al., 2011; Razansky et al., 2012; Sun et al., 2018), which are applied in drug development, discovery, delivery, as well as therapy monitoring. Combining with miniaturization and endoscopy technology, optical microscopic imaging can even be applied to high-resolution imaging *in vivo* (Jabbour et al., 2014; Zong et al., 2017). In addition, other emerging OI techniques, such as fluorescence lifetime imaging and multiphoton imaging, can also be used for drug discovery and screening (Konig et al., 2006; Kumar et al., 2011).

**Figure 3:**
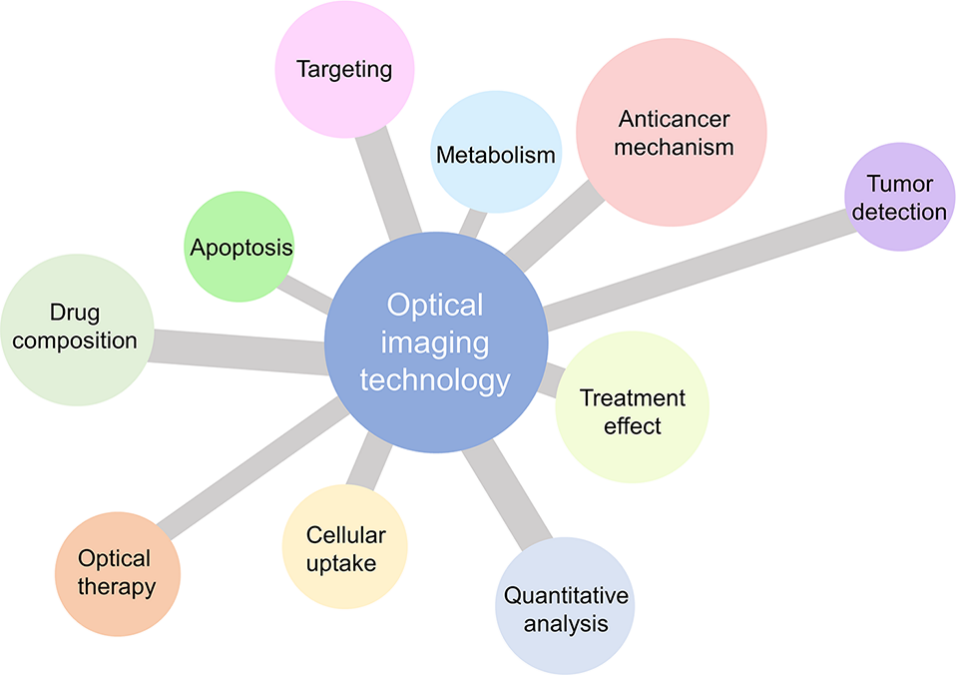
Application potential of optical microscopic and macroscopic imaging for natural products as cancer therapeutics.

In summary, OI technology, including both the optical microscopic imaging and macroscopic imaging techniques is a very important tool in the field of medicinal chemistry. We believe that the power of optical microscopic and macroscopic imaging will become a major boost for NPs as cancer therapeutics (**Figure 3**).

## Author Contributions

TY, QZ and XC designed the structure. TY and NW summarized the part of optical microscopic imaging. TY, LW, and HC summarized the part of optical macroscopic imaging. TY and QZ prepared the manuscript. TY, XX and XC modified the manuscript. TY and QZ contributed equally to this work.

## Funding

This work was supported in part by the National Key R&D Program of China under Grant No. 2018YFC0910600, the National Natural Science Foundation of China under Grant Nos. 81627807, 11727813, 81571725, 81701853, 81871397, 91859109, 81660505, the Fok Ying-Tong Education Foundation of China under Grant 161104, the Program for the Young Top-notch Talent of Shaanxi Province, the Research Fund for Young Star of Science and Technology in Shaanxi Province under Grant No. 2018KJXX-018, the Best Funded Projects for the Scientific and Technological Activities for Excellent Overseas Researchers in Shaanxi Province (2017017), the Natural Science Basic Research Plan in Shaanxi Province of China under Grant Nos. 2018JM7072, 2019JQ-201, 2019JQ-045, and Fundamental Research Funds for Central Universities (JB181203, JB191201, JB191209).

## Conflict of Interest

The authors declare that the research was conducted in the absence of any commercial or financial relationships that could be construed as a potential conflict of interest.
